# The Right to Rehabilitation for People With Dementia: A Codesign Approach to Barriers and Solutions

**DOI:** 10.1111/hex.70036

**Published:** 2024-09-24

**Authors:** Natasha Layton, Catherine Devanny, Keith Hill, Kate Swaffer, Grant Russell, Lee‐Fay Low, Den‐Ching A. Lee, Monica Cations, Helen Skouteris, Claire MC O'Connor, Taya A. Collyer, Barbara Barbosa Neves, Nadine E. Andrew, Terry Haines, Velandai K. Srikanth, Alan Petersen, Michele L. Callisaya

**Affiliations:** ^1^ Rehabilitation Ageing and Independent Living (RAIL) Research Centre Monash University Melbourne Victoria Australia; ^2^ National Centre for Healthy Ageing Melbourne Victoria Australia; ^3^ Peninsula Clinical School, School of Translational Medicine Monash University Melbourne Victoria Australia; ^4^ University of South Australia Adelaide South Australia Australia; ^5^ Department of General Practice, School of Public Health and Preventive Medicine Monash University Melbourne Victoria Australia; ^6^ Faculty of Medicine and Health University of Sydney Sydney New South Wales Australia; ^7^ College of Education, Psychology and Social Work Flinders University Adelaide South Australia Australia; ^8^ Health and Social Care Unit, School of Public Health and Preventive Medicine Monash University Melbourne Victoria Australia; ^9^ School of Psychology University of New South Wales Sydney New South Wales Australia; ^10^ HammondCare, Centre for Positive Ageing Sydney New South Wales Australia; ^11^ Neuroscience Research Australia Sydney New South Wales Australia; ^12^ Sydney Centre for Healthy Societies University of Sydney Sydney New South Wales Australia; ^13^ School of Public and Allied Health Care Monash University Melbourne Victoria Australia; ^14^ Peninsula Health Melbourne Victoria Australia; ^15^ Sociology, School of Social Sciences Monash University Melbourne Victoria Australia; ^16^ Menzies Institute for Medical Research Hobart Tasmania Australia

**Keywords:** allied health occupations, dementia, general practice, healthcare disparities, lived experience, reablement, rehabilitation, stigma

## Abstract

**Introduction:**

People with dementia of all ages have a human right to equal access to quality health care. Despite evidence regarding its effectiveness, many people living with dementia are unable to access rehabilitation for promoting function and quality of life. Conducted in Australia, this study was designed to (1) explore barriers to access to dementia rehabilitation and (2) identify solutions that improve access to rehabilitation.

**Methods:**

People living with dementia (*n* = 5) and care partners (*n* = 8) and health professionals (*n* = 13) were recruited nationally. Experience‐based codesign across three virtual workshops was used to understand barriers and design solutions to improve access to rehabilitation treatments. Socio‐ecological analyses, using the Levesque Access to Health care framework, were applied to findings regarding barriers and to aid selection of solutions.

**Results:**

There was high attendance (92.3%) across the three workshops. Barriers were identified at a user level (including lack of knowledge, transport, cost and difficulty navigating the health, aged care and disability sectors) and health service level (including health professional low dementia knowledge and negative attitudes, inequitable funding models and non‐existent or fragmented services). Solutions focused on widespread dementia education and training, including ensuring that people with dementia and their care partners know about rehabilitation therapies and that health professionals, aged care and disability co‐ordinators know how to refer to and deliver rehabilitation interventions. Dementia care navigators, changes to Australia's public funding models and specific dementia rehabilitation programmes were also recommended.

**Conclusions:**

Barriers to accessing rehabilitation for people with dementia exist at multiple levels and will require a whole‐community and systems approach to ensure change.

**Patient or Public Contribution:**

People with living experience (preferred term by those involved) were involved at two levels within this research. A Chief Investigator living with dementia was involved in the design of the study and writing of the manuscript. People with living experience, care partners and service providers were participants in the codesign process to identify barriers and design potential solutions.

## 1 Introduction

Globally, more than 55 million people have dementia [1]. Over 415,000 people currently live with dementia in Australia, and this is estimated to increase to 1.2 million people by 2056 [2]. Dementia affects memory and a wide range of other cognitive, behavioural, psychological and physical functions that result in disability. This can include a loss of ability to participate in everyday, social and occupational activities.

There is evidence that nonpharmacological treatments can reduce disability, including those caused by dementia, by improving function and quality of life, especially if provided in the mild to moderate stages of dementia [3, 4, 5, 6, 7, 8]. These interventions have been described as ‘dementia rehabilitation’ by the World Health Organisation [9]. Australian clinical practice guidelines also recommend that people with dementia be referred to allied health professionals to maintain or improve function for as long as possible [10]. For example, therapy delivered by occupational therapists, physiotherapists, psychologists and speech pathologists can improve cognition [3, 8], physical function [4], communication [6, 11], every day activities [6, 12], mental health [11] and reduce falls [13]. Additionally, many of these treatments reduce care partner stress, a major driver of admission to aged care [14].

However, people living with dementia are often unable to access evidence‐based rehabilitation. Health professionals, and others, tend to believe that people with dementia are unable to engage in or benefit from rehabilitation treatments (therapeutic nihilism) [15, 16], likely stemming from low levels of dementia knowledge [15, 16]. People with dementia and their care partners have reported difficulties finding and navigating rehabilitation services [17] and report needing more information and support [18]. From a human rights perspective, access to rehabilitative healthcare should be within reach of all, especially where evidence for effective interventions exists [3, 4, 6, 8].

Contemporary thinking about consumer‐centred access to rehabilitation considers the fit between the health system, organizations, health providers and the characteristics and expectations of clients [19]. In recognition of the fact that individuals who seek, use or provide services may not be in positions of power, methodologies are required that allow research to be conducted ‘with’ and not ‘on’ consumers [20]. Sometimes described as coproduction or emancipatory practice, codesign is a conceptual and methodological approach that values the voices of service users and other stakeholders [21]. codesign within research benefits research processes and outcomes [22]. To date, codesign methods have not been applied to examine the barriers to rehabilitation access for people with dementia, or to generate solutions.

Therefore, the aim of this study was to determine barriers and codesign potential solutions to improve access to dementia rehabilitation with various stakeholders, including people living with dementia, in Australia. This is phase one of a larger study funded by the Medical Research Future Fund Australia (#2015947) aiming to improve access to rehabilitation for people living with dementia in the community. Codesign informed both the research design (one of the chief investigators lives with dementia) and the selection of research methods.

## 2 Methods

### 2.1 Study Design and Frameworks

Data were collected through three virtual workshops with a series of supporting activities intended to empower participants and thereby to enact codesign. A commitment to engagement throughout the project was underpinned by the Australian Department of Health Stakeholder Engagement Framework [23]. Experience‐based codesign [24] was selected as it provides a structured process for the provision of an equal voice and user experience of a range of stakeholders. It includes five stages: set up, gather the experience, understand the experience, improve the experience, monitor and maintain the experience. Levesque's Conceptual Framework of Access to Health was used as an analytic coding frame to guide analysis of the barriers and solutions [25]. The framework describes both healthcare service and interrelated user‐level dimensions to accessibility, as well as spanning the initial period of the user perceiving the need for rehabilitation to remaining engaged with services overtime. A summary of the dimensions is as follows: ‘Perception of Needs’ and ‘Desire for Health Care’ cover individual's *ability to perceive* rehabilitation services and the degree to which the service is known to exist (*approachability*). ‘Health Care Seeking’ is the individual's *ability to seek* rehabilitation services and the *appropriateness and acceptability* of services. ‘Health Care Reaching’ focuses on the ability of the person to reach a service, and whether on the service side it can be reached in a timely way (*availability and accommodation*). ‘Health Care Utilization’ is the individual's *ability to pay* and the cost of the rehabilitation service (*affordability*). ‘Consequences of Accessing Health Care’ considers the ability of the individual to engage in rehabilitation, and the extent to which services meet the needs of individuals (*appropriateness*).

Ethics approval was obtained from the Monash University Human Research Ethics Committee (Project #35555). All workshop attendees provided written or verbal consent.

### 2.2 Participants and Recruitment

Purposeful sampling aimed to establish two stakeholder groups of people residing in Australia (Table 1). Living experience experts were recruited via advertising through national and international dementia organizations. Advertisement encouraged participation from people with dementia and care partners of different ages, cultural and linguistic backgrounds and both rural and urban areas, but people had to be residing in the community. Representatives from both organizations passed interested people's names on to the research team, who then provided Explanatory Statements and gathered Consent Forms from individuals who elected to participate. Consistent with ethical requirements for this population, Explanatory Statements were in plain language and large font, and the research team provided information in each individual's preferred formats (mail or email) and were available to talk through content at times convenient to the individual and with a care partner if preferred. Professionals known to be experienced in working with people with dementia were identified through various sources (Table 1). A gift voucher of $150 per workshop meeting (acknowledging preparation and workshop meeting time) was offered to both groups. Desired numbers were reached on the basis of this recruitment strategy.

**Table 1:** Stakeholder mix and recruitment source for the codesign workshops.

Workshop groups	Makeup of the codesign workshop	Recruitment source and methods
Group A (living experience experts)	People living with dementia and their care partners	Dementia Australia (email) Dementia Alliance International (email)
Group B (health professionals)	Health service managers, general practitioners, practice nurses, geriatricians, neurologists and allied health professionals	Advertisements though primary health network newsletters. Personal contact though research and clinical networks
	Representatives from partners' organizations: Dementia Australia, Primary Health Care Networks and national allied health professional associations (e.g., Australian Physiotherapy Association, Occupational Therapy Australia)	Researchers contacted the partners' organizations, and organizations identified the most relevant person to participate

### 2.3 Codesign Procedure

#### 2.3.1 Stage 1: Set‐Up

A steering committee was drawn from the Chief Investigator panel of the larger project and included a diverse range of backgrounds, including living experience, research, health professions, management and policy. A number of the steering committee members had published research regarding the evidence for dementia rehabilitation and the need for better access [6, 15, 16, 18, 26]. This work provided extensive background to both the need for the project and for the workshops.

MC, NL and CD ran the workshops and also created the codesign protocol under direction from a subset of the steering committee, generating topic guides and questions based upon the Experience‐Based Codesign Toolkit [24]. The aims and broad structure for each workshop were provided in the preworkshop Briefing Packs, and workshop participants were invited to shape these as part of the regular ‘terms of engagement’ check at the commencement of each workshop. MC, ML and CD hold backgrounds in occupational therapy and physiotherapy and have worked in various contexts with people living with dementia. From a reflexivity perspective, workshop conveners were familiar with the organizations from which participants had been recruited, and did not have prior relationships with any of Group A. Any relationships with members of Group B. were collegiate, that is, not funded relationships or relationships involving power discrepancies.

#### 2.3.2 Stages 2‐4: Gather, Understand and Improve the Experience Workshops

Workshops were run via online video conferencing (Zoom). To support people living with dementia and their care partners, an initial ‘meet and greet’ workshop was offered to introduce the codesign process, help people gain confidence to speak up, practice and identify any assistance needed in using Zoom and to identify preferences about the format and timeframe for meeting reminders and reading materials.

Both groups then participated in three workshops, with permission gained to record each workshop for the purposes of later transcription. Workshop 1 ‘gather the experience’ and Workshop 2 ‘understand the experience’ were 3 weeks apart to provide time to read briefing materials and respond to workshop summaries whilst being close enough together to maintain momentum. These workshops were run separately for each group. Three months later, Workshop 3 ‘improve the experience’ was run once with both groups in attendance. A 3‐month gap enabled the researchers to collate all information and discuss implementation of solutions with partners. All workshops ran for 90 min and involved breakout groups to allow everyone the chance to speak. Each workshop was facilitated by three members of the research team experienced in facilitating workshops and working with people living with dementia (NL, CD and MC). (Supporting Information S1: Table S1 summarizes the content of each workshop).

To support each workshop, briefing notes (see Supporting Information S1: Table S2 for further details), PowerPoint decks and summaries of prior workshops were provided via email or mail, according to participant preference. Illustrations were commissioned, reviewed by the advisory group and utilized to illustrate barriers and pathways to rehabilitation. Table 2 shows how the principles of engagement drawn from the Department of Health Stakeholder Engagement Framework [23] were enacted during and between workshops.

**Table 2:** Underpinning principles of engagement drawn from the Department of Health Stakeholder Engagement Framework [23].

Terms of Engagement	Narrative provided within workshops to explain the principles and guide Engagements	Examples of how the principles were shared and met
Purposeful	We will make a difference. We will work on access to important therapies and supports for people living with dementia.	Briefings notes on the importance and aims of the project.
Inclusive	At every stage, we will include different professions and different groups, including people living with dementia and care partners. We will provide ways for everyone to contribute. We will be open to feedback about doing this better.	Terms of Engagement presented in a slide at each workshop. Advertisement encouraged a diverse range of people. ‘Meet and greet’ sessions for support and to understand preferences. Multiformats of communication, including one‐to‐one support. Inviting feedback via email or phone between sessions. Three meeting facilitators to ensure that help was available. Asking if everyone is ok during workshops.
Timely	The government has funded this research now. Workshops will be arranged to guide the research at important steps. We will provide reading time and hold our workshops at times that suit most people.	Consultation to establish preferred times for workshops. Invited feedback at workshops on timings.
Transparent	We will provide information so everyone can join in and share ideas. We will clearly explain our codesign process and ask how it is going. We will clearly describe the context and constraints of the project.	Writing perspectives on a PowerPoint for all to see and comment on during the workshops. Inviting feedback on the process and content at each workshop. Providing summaries for feedback to all after each workshop. Presenting the context and parameters for solutions that could be chosen for implementation in the project.
Respectful	Codesign is a two‐way process. Everybody's perspectives will be equally valued.	Presenting the terms of engagement via PowerPoint slide at commencement of every workshop. Undertaking to report all ideas. Allowing time for all participants to speak. Introducing attendees and acknowledging experience. Following dementia language guidelines [27].

### 2.4 Data Analysis

The workshop audio transcriptions and PowerPoint annotations were summarized in Word using tables by one researcher (NL), member‐checked by the other two researchers (CD and MC) and then circulated to all workshop attendees for verification. These included summaries of the discussion and key quotes from each workshop. Attendees were asked to check the summaries to ensure that no information had been missed and add to the summaries if they had any further comments. MC and NL utilized these summaries to independently code (1) barriers and (2) solutions descriptively into the predetermined codes of Levesque's access to healthcare dimensions [25]. These were then discussed, and NL generated the final codes that were then member‐checked by MC.

## 3 Results

### 3.1 Participants

Thirteen living experience experts (five individuals with dementia and eight care partners) from across six Australian states, including six rural and seven metro areas, participated in the workshops. Thirteen health professionals across ***three Australian states, including one rural area***, participated, including representatives of three allied health professional bodies, a national dementia organization, two managers and health professionals working with people with dementia (both private and public health). Their backgrounds included neurology (1), geriatrics (1), general practice (GP) (2), physiotherapy (1), occupational therapy (3), speech pathology (3) and nursing (2).

### 3.2 Participation in Workshops

All of group A elected to attend the optional ‘Meet and Greet’ workshop. Participation rates in Workshops 1 and 2 were 100% for both groups. For Workshop 3, 12/13 of living experience experts and 11/13 of the professionals group participated due to the inability to make the appointment rather than not wanting to attend.

### 3.3 Workshop Findings

The Access to Healthcare Framework was found to be appropriate to the data generated, with no data unaccounted for.

#### 3.3.1 Barriers to Accessing Rehabilitation

**Figure 1:**
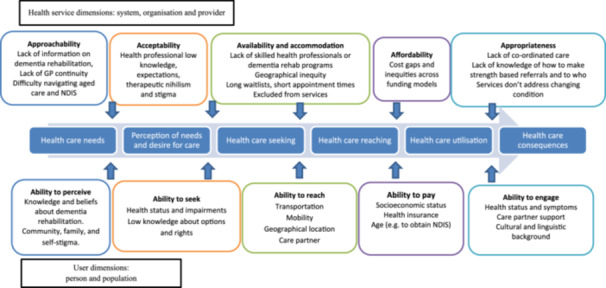
Barriers to accessing rehabilitation using the Access to Healthcare Framework.

Barriers to accessing rehabilitation were identified by both groups. Figure 1 shows these barriers organized into user and healthcare service dimensions [25]. The most frequently discussed barriers are described below (components of the Levesque framework are in bold).

A key factor contributing to the **inability to perceive** the need for rehabilitation and **seek it** was that the person with dementia and their care partners did not know about rehabilitation therapies. *I need to know what can I do to make my life better. I was never able to find someone to help me*—*even though I was trying* [Group A ***person living with dementia***]. This was compounded by providers and organizations (dementia or carer organizations) not having information readily available about the range of therapies that allied health professionals can offer (**approachability**).

Healthcare **seeking** was seen as difficult, as attendees described that GPs and specialists often held negative attitudes and beliefs (**acceptability**) about the ability of people with dementia to engage or improve with therapy, and therefore, referrals were not made. This was attributed to lack of dementia training: *… it sounds ridiculous but none of us (GPs) have had much education in this space.* [Group B]. Low knowledge, paternalism and therapeutic nihilism were described as issues across all professionals (medical specialists, allied health professionals, aged care assessors, disability co‐ordinators and case managers). *They think that dementia means someone is tucked away and can't do anything anymore* [Group A ***care partner***]; *They want to keep people safe, this leads to limited opportunities and activities*; *Assessment teams for My Aged Care* [Service for accessing Australian Government‐funded aged care services] *have no knowledge of benefits of allied health for people with dementia* [both Group B]. Policies that excluded people with dementia from some rehabilitation services were described as a barrier. **Seeking** and applying for services were commonly described as *hard to navigate* [Group A ***person living with dementia***], and cognition and communication problems made **seeking** services through MyAgedCare (Australian government aged care services) or the National Disability Insurance Scheme [Australian government scheme providing funding for people with a disability if under 65 years] even more difficult.

Barriers to **reaching** services were present at both the user and service levels. The lack of health provider knowledge and acceptability meant that there was often no one delivering rehabilitation for people with dementia *Somebody's got to be out there delivering the goods* [Group A]. **Availability** was noted as worse in rural areas, where there were fewer professionals trained in dementia. Inability to see the same general practitioner and short appointment times (**accommodation**) were thought to contribute to a lack of timely referrals, with one attendee concluding *Fast medicine*—*it takes time to know the person and their needs, general practitioners have little time and many other issues to address.* [Group B]. If rehabilitation services did exist, lack of transport (people with dementia often give up driving), poor mobility or reliance on a care partner were identified barriers to **reaching** services.

The **ability to pay** for private allied health professionals was seen as a barrier, with few options available in the public system. Funding through Medicare Chronic Disease Management Plans and Team Care Arrangements (initiated by general practitioners) were seen as a short‐term option, with only five allied health professional sessions funded per year, and these did not cover full costs, especially where home visits were required. Services delivered through Home Care Packages (Commonwealth government‐funded support to help people remain at home for longer) also did not cover the full costs. These issues *… could be addressed if My Aged Care funded enough allied health sessions, and if allied health services were prioritized. The [National Disability Insurance Scheme], for younger people, represents a pathway that enables better access, which is helping to bring allied health into this field.* [Group B]

Even if a person with dementia was able to receive a home care package or reach an allied health professional, services were frequently seen as not fitting all the needs of people with dementia (**appropriateness**) and had long wait times. Attendees from both groups described that services did not offer therapies that supported the person to *continue to do activities*; rather, they implemented services that only *did things for* the person, leading to greater disability and lower quality of life. Support and allied health services were seen as *fragmented,* with no one to co‐ordinate care or address issues that arose due to the progressive nature of dementia, often relying on an assertive and knowledgeable care partner to navigate services and advocate for them.

#### 3.3.2 Understanding and Improving Solutions

**Figure 2:**
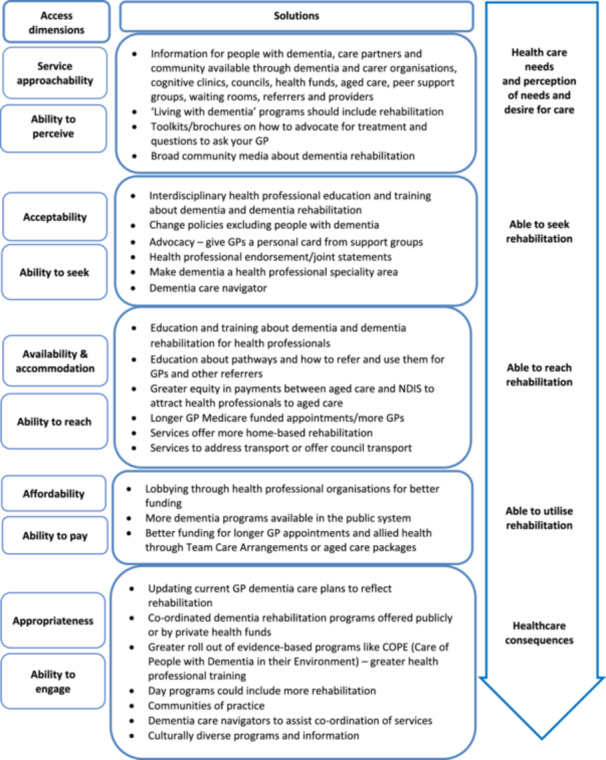
Solutions at each level of access dimensions.

Figure 2 shows the suggested solutions according to the different dimensions of access.

#### 3.3.3 User Dimensions: People With Dementia and Care Partners

Suggestions as to how to support people with dementia (and care partners) access rehabilitation were focused mainly around providing better resources and education and having access to a dementia navigator. Some suggestions targeted more than one domain, for example, the need for a dementia navigator to improve a person's ability to seek and engage in services as dementia progressed and needs changed.

Attendees from both groups recommended that information on dementia rehabilitation (including how to ask for it and pathways to access it) should be available through dementia organizations and integrated into currently available dementia guides, booklets and programmes geared towards educating consumers and families about living with dementia (**ability to perceive and seek**). Self‐assessment apps, peer‐to‐peer support, word of mouth, GP waiting rooms, cognitive clinics, day programmes, council websites and other locations where families could look up resources were seen as important points through which to access such resources.

The need for a support person to help **seek and engage** with services was seen as key by the two groups. *Understanding what people value, and what they have done in their life, is key knowledge too. But things can be missed without a proactive advocate or care partner and without a care plan.* [Group A ***care partner***]. Existing initiatives such as case managers and co‐ordinators from the disability sector were thought to be beneficial, but they also needed specific training in dementia. Navigators from other conditions were used as examples: *Navigating services is a huge issue, and with cancer care nurses this works well*. [Group A ***person living with dementia***] *Patients love to have one person to call* [Group B].

#### 3.3.4 Health Service Dimensions: Provider, Organizations and Health Systems

At the health service level, key solutions included greater education and training for both referrers and providers, greater and more equitable funding for general practitioners and aged care and availability of specific dementia rehabilitation programmes. Some suggestions covered more than one dimension of access.

Education and training were suggested to improve **acceptability, availability and appropriateness** of services. Breaking down stigma that prevents the possibility of rehabilitation for people living with dementia and increasing the number of knowledgeable health professionals were seen as essential. *To have a successful pathway, we need people at the other end*—*who are accepting the referrals*—*to say ‘yes’. We need to work with agencies and intake workers*—*both public and private health services*—*to make sure that people with dementia are not being unfairly denied support with rehabilitation and reablement.* [Group B]. Although general education courses about dementia are available, groups identified gaps in education about dementia rehabilitation at undergraduate and post‐graduate levels. Both groups described some key considerations when designing this education and training (Box 1).

Box 1 **Content and format to include in education and training on dementia rehabilitation for health professionals, people with dementia, care partners and the general community.**

**Content**

- Incorporate strength‐based approaches and ways to enable positive risk taking for quality of life;
- Highlight that people with dementia are not all the same and need individual person‐centred treatments;
- Inform on treatments that support people to continue to do activities rather than just ‘do for them’;
- Include individual case studies, positive stories and pathways that feature people living with dementia;
- Clearly define dementia rehabilitation using the World Health Organisation definition and differentiate it from short‐term rehabilitation for other acute conditions;
- Acknowledge dementia as a progressive condition with changing needs;
- Highlight the scope of allied health. Speech Pathology is more than swallowing (e.g., communication interventions and strategies); Occupational Therapy is more than home modifications (e.g., strategies to maintain activities); and physiotherapy is more than improving balance after a fall (e.g., exercise prescription to slow cognitive decline);
- Include how to advocate for access to treatments; and
- Include a simple sheet describing what people can do for themselves for their mind and body (to provide information to people with dementia and the general community).
**Format and Delivery**
- Include people living with dementia in delivering the training;
- Training should be interdisciplinary;
- Make it interesting and intellectually challenging;
- A community of practice to enable learning, mentorship and networking; and
- Ensure simple, easy‐to‐read information and a range of formats and languages if available (to provide as education for people with dementia and the general community).

General practitioners themselves explained they need education and support to refer people for rehabilitation. *There are pathways written for GPs but we need more info on how to use them for individual patients and to find local services* [Group B]. It was suggested that general practitioners need to know how to assess for rehabilitation, write strength‐based referrals and understand services in their area. Communication tools and checklists leading to an action plan that included rehabilitation were suggested. They could ask *How socially connected are you? What are you having trouble with? What do you want to try to continue to do?* [Group B]. Dementia Training Australia was mentioned as an organization that trains general practitioners and has existing dementia care plans that could be modified. It was acknowledged that it is difficult for general practitioners to be an expert across all the different common medical conditions, and that an additional option might be to train practice nurses to assess and refer to the appropriate allied health professionals.

Attendees noted that education alone was not enough and that funding changes were required to address **affordability** of services. General practitioners need longer, and potentially multiple, consultations. At a professional association level, it was suggested that an allied health strategy, bringing together all professional bodies, would provide a bigger voice to advocate to government and private health insurance providers for greater allied health funding.

Specialist dementia rehabilitation programmes were suggested to provide a direct pathway and more skilled and **appropriate** services through improved co‐ordination of care such as those available for knee arthritis or pulmonary rehabilitation. These could be funded by private insurance companies or delivered by public rehabilitation and community health centres. Attendees also mentioned that many other stakeholders had a role to play in facilitating access to rehabilitation within the Australian system. Managers and care navigators across aged care and disability sectors were identified as needing education so that they could ensure that when people with dementia did receive funding, they could recommend and provide **appropriat**e services for changing needs.

## 4 Discussion

Rehabilitation is recommended by the World Health Organisation for people living with dementia in order to maximize function and participation [9]. However, rehabilitation is often not available or offered. To date, few studies have examined barriers and solutions to accessing dementia rehabilitation, with most focusing on either the user [18, 28] or healthcare level [17, 26] alone. This study was designed in partnership with a researcher with lived experience of dementia and conducted through three codesign workshops with key stakeholders inclusive of living experience experts, care partners and health stakeholders. The study endeavoured to achieve quality stakeholder engagement and to action experience‐based codesign through use of the Australian Department of Health Stakeholder engagement framework and the Experience‐Based Codesign Toolkit for Australia [23, 24]. In this study, multiple barriers were found to accessing rehabilitation that resulted from interactions between user and health service characteristics. The main suggested solutions to assist people with dementia and their care partners were greater provision of dementia rehabilitation education/resources and dementia care navigators. At the healthcare level, education and training across professionals were recommended, along with greater advocacy for better funding, and specialized dementia rehabilitation programmes.

A key barrier was that little, if any, information about dementia rehabilitation is provided to the person or care partner, limiting their ability to perceive the benefits of, or seek, treatments. This is in line with a prior study that highlighted that people with dementia are rarely told about, or referred to, rehabilitation services [18]. This is not surprising as it was noted that there is little training on dementia at an undergraduate level for medical or allied health professionals, a fact likely to be a key driver of nihilistic attitudes about the ability of people with dementia to benefit from rehabilitation [15, 16]. Solutions at the user level included providing more information through Dementia Australia's ‘Dementia Guide’ and ‘Living with Dementia’ programmes. This appears feasible, although there is currently little evidence to show that information alone will change perceptions of need, or the ability to seek appropriate treatments, and more individualized interventions may be required [29].

At the healthcare service level, services were described as excluding people with dementia, or only providing services that do things for a person, rather than empowering them to be independent. At this level, solutions included providing education and training across health, dementia organizations, aged care and disability sectors and in university undergraduate programmes. Education and training should incorporate strength‐based approaches and ways to enable positive risk taking for quality of life [30]. Policy change may also be necessary, with the Royal Commission into Aged Care in Australia stating that rehabilitation is a priority to optimize independence and well‐being for all older people [31]. Online general dementia education courses have been successful in changing knowledge on a national and worldwide scale [32], but practical or experience‐based learning is likely to be needed to improve practical skills. For example, an evidence‐based dementia programme that involves training occupational therapists has been found to be successful in supporting people with dementia with everyday activities and was successfully implemented in an Australian setting [33]. A tiered model could be adopted in Australia, similar to Alzheimer's Scotland [34], where there are more specialized therapists who complete comprehensive dementia training, and then a more accessible on‐line model to target a wider number of professionals to raise awareness and ensure that appropriate onward referrals are made. Work is also underway to create dementia education training standards in Australia [35], which will aid in the design of such courses.

GPs and specialists needed to know how to make strength‐ and goal‐based referrals as well as having clear local pathways to refer to specific dementia rehabilitation programmes or professionals. With limited specialist cognitive clinics in Australia and their focus on diagnosis, GPs were seen by workshop attendees as key referrers and co‐ordinators of care. Prior studies have found that decision support software and practice‐based workshops [36] or practice‐based academic detailing [37] have the potential to improve GPs' ability to detect dementia. Dementia Training Australia runs GP dementia training workshops across Australia. This may provide a way to incorporate education on dementia rehabilitation and referrals that could be coupled with activities to review performance. This could be designed to meet GP annual continuing professional development requirements. However, training relies on people enrolling, and therefore, undergraduate content will also be important for the future workforce. Most of Australia's Primary Health Networks are working with the HealthPathways resource to consolidate information on the early management and care of people living with dementia [38]. However, advocacy is needed to ensure that pathways are used by GPs and that they include information and options for referral to allied health and rehabilitation.

Stakeholders noted that education and training would not solve all problems. To be able to successfully seek, reach and engage in services, such services need to be available, and people with dementia and care partners need to be advised of them. People with dementia may also need assistance to navigate the health and social sector, not just at the time of diagnosis, but as disability progresses. Furthermore, these services will need to be available as the person's condition progresses (adequacy of services), and reach people in rural areas, people from low socioeconomic or different cultural backgrounds and those without transport or who have difficulty going out due to mobility or fatigue. Dementia navigators and case managers exist in other countries, and have been found to have varying impact; however, differing models and different qualifications of navigators make it hard to determine effective components [39, 40, 41]. Longer term case management models were found to have the strongest evidence for improving outcomes [41]. However, lobbying for funding in Australia will be required. Other solutions included advocacy for better funding for longer general practice consults, closing funding inequities between current disability and aged care services for allied health, greater equity of services in rural areas and having specific dementia rehabilitation programmes (including those delivered at home) to provide a clear pathway, with recognition that due to the progressive nature and changing needs, people may need intermittent or ongoing involvement with such programmes.

In summary, this study highlights that no one solution would improve access to rehabilitation for people with dementia. A number of solutions would require policy and funding changes at a State or Commonwealth government level. Attendees in the workshops concluded that there was currently insufficient information available on dementia rehabilitation and the scope of therapies that allied health professionals can offer. Although realizing that improving knowledge may not change availability of services in the short term, it was an important first step in raising awareness and providing a ground swell of advocacy to facilitate change. Education across the community and professions will become the focus of a second stage of this project and will be guided by stage four of the Evidence‐Based Codesign toolkit for Australia ‘monitor and maintain the experience’.

The strengths of the study include use of the Eevidence‐Based Codesign process based on a published method [24] and terms of engagement [23]. The ‘meet and greet’ session solved potential difficulties for future workshops. The high degree of workshop attendance demonstrated commitment to the process. The use of separate workshops for living experience experts and professionals was one way to manage numbers, but also enabled a cohort effect to build in joining together with shared perspectives in the ‘gather’ and ‘understand’ phases. Separating stakeholder groups was also a way to manage power differences and to build confidence. There were also study limitations. The codesigners were from Australia, and so the solutions were naturally focused on Australia and may not be suitable for other countries, although barriers such as stigma are noted internationally [42]. The combined larger group of 23 in the final workshop was positive in that both groups interacted to discuss solutions, but despite utilizing three breakout rooms, discussion was more challenging. The use of videoconferencing may have excluded people without internet or computer skills. However, it did allow for participation across Australia, including rural areas and for those who had difficulty with transport or mobility. A further limitation was that the scope was confined to community settings, and therefore set boundaries on a comprehensive exploration of options in residential aged care and hospital settings. Finally, we did not collect information on age, gender, the stage and type of dementia or whether people with dementia had accessed rehabilitation services (and the type) or not, which may have influenced the barriers and solutions that were designed.

## Author Contributions

**Natasha Layton:** conceptualization, investigation, funding acquisition, writing–original draft, methodology, writing–review and editing, formal analysis, project administration. **Catherine Devanny:** conceptualization, writing–original draft, methodology, writing–review and editing, project administration, investigation. **Keith Hill:** conceptualization, investigation, funding acquisition, writing–review and editing. **Kate Swaffer:** conceptualization, investigation, funding acquisition, methodology, writing–review and editing. **Grant Russell:** conceptualization investigation, funding acquisition, writing–review and editing. **Lee‐Fay Low:** conceptualization, investigation funding acquisition, writing–review and editing. **Den‐Ching A. Lee:** writing–review and editing, project administration, investigation. **Monica Cations:** conceptualization, investigation, funding acquisition, writing–review and editing. **Helen Skouteris:** conceptualization, investigation, funding acquisition, writing–review and editing. **Claire MC O'Connor:** conceptualization, investigation, funding acquisition, writing–review & editing. **Taya A. Collyer:** conceptualization, investigation, writing–review and editing, funding acquisition. **Barbara Barbosa Neves:** conceptualization, investigation, funding acquisition, writing–review and editing, methodology. **Nadine E. Andrew:** conceptualization, investigation, funding acquisition, writing–review and editing. **Terry Haines:** conceptualization, investigation funding acquisition, writing–review and editing. **Velandai K. Srikanth:** conceptualization, investigation, funding acquisition, writing–review and editing. **Alan Petersen:** conceptualization, investigation, funding acquisition, methodology, writing–review and editing. **Michele L. Callisaya:** conceptualization, investigation, funding acquisition, writing–original draft, methodology; writing–review and editing, project administration.

## Ethics Statement

Ethics approval was obtained from the Monash University Human Research Ethics Committee (Project No. 35555).

## Conflicts of Interest

The authors declare no conflicts of interest.

## Supporting information

Supporting information.

## Data Availability

The data are not publicly available due to privacy and ethical restrictions. Please contact the corresponding author with requests for data.
