# Supplementary material for: The Right to Rehabilitation for People With Dementia: A Codesign Approach to Barriers and Solutions
Source: Health Expect. 2024 Sep 24;27(5):e70036. doi: 10.1111/hex.70036 (PMC11422665; doi:10.1111/hex.70036)
Supplement: Supplementary file 1 — Supporting information. [file HEX-27-e70036-s001.docx]

**Supplementary Table 1** Co-Design workshop agendas

| **Panels** | **Agenda** |
| --- | --- |
| Optional ‘Meet and Greet’ meeting (Group A only) | One week prior to the first workshop, a half hour Zoom session was offered to introduce group A to each other, familiarise everyone with zoom, determine workshop preferences and help make people feel comfortable to speak up. |
| Workshop 1 ‘gather the experience’  (Group A and B* separately) | - Welcome, introductions, terms of engagement - Overview of project, co-design process, context and constraints - Overview of rehabilitation and evidence - Overview of literature on known barriers (stigma, knowledge, disempowerment, lack of pathways) - Breakout groups-living experience and practice knowledge - Checking in everyone is ok   Engagement Strategies: live annotation of PowerPoint questions; illustrated persona (Lee on his journey) |
| Workshop 2 ‘understand the experience’  (Group A and B separately) | - Welcome and re-introductions, terms of engagement, context - Workshop 1: any comments? - Workshop 2 understand the experience  Breakout groups: imagining solutions (e.g. person, GP, allied health, pathways) - Checking in everyone is ok   Engagement Strategies: live annotation of PowerPoint questions; illustrated personas (what did/ did not work for Lee; pathways for Rose) |
| Workshop 3 ‘improve the experience’  (Group A and B together in single workshop) | - Welcome & introduction to our combined co-designers - Review project aims and terms of engagement - Reflections and feedback on process so far - Update from the project team on discussions with partners about rolling out solutions - Breakout groups: refining solutions - Next steps   Engagement Strategies: live annotation of PowerPoint questions; illustrated personas of suggested solutions |

Group A - Living experience experts; Group B – Health professionals

**Supplementary Table 2** Summary of Materials provided to Co-design Workshop Participants

| **Briefing Pack 1 (provided prior to Workshop 1)** | |
| --- | --- |
| **Briefing Note** | Brief plain language summary of the evidence regarding the right to rehabilitation for people with dementia:   - What does the evidence tell us? - What problem needs solving? - What are the known barriers? - Aims of project - Question for Workshop 1: How might we improve access to rehabilitation for people with dementia? - Examples of rehabilitation for people with dementia (one page) - References |
| **Briefing Pack 2 (provided prior to Workshop 2)** | |
| **Summary of Workshop 2** | Summary of workshop 1 from both groups’ discussions |
| **Briefing Note** | Plain language summary including what we know from Workshop 1  Preparation for Workshop 2   - workshop 2 PowerPoint slide pack - Existing resources: for people with dementia, their families and care partners, referrers, allied health professionals |
| **Briefing Note 3 (provided prior to Workshop 3)** | |
| **Summary of Workshop 2** | 1. The problem we are tackling 2. Summary of workshop 2 from both groups discussion 3. Example of illustrations used in Workshops and materials:   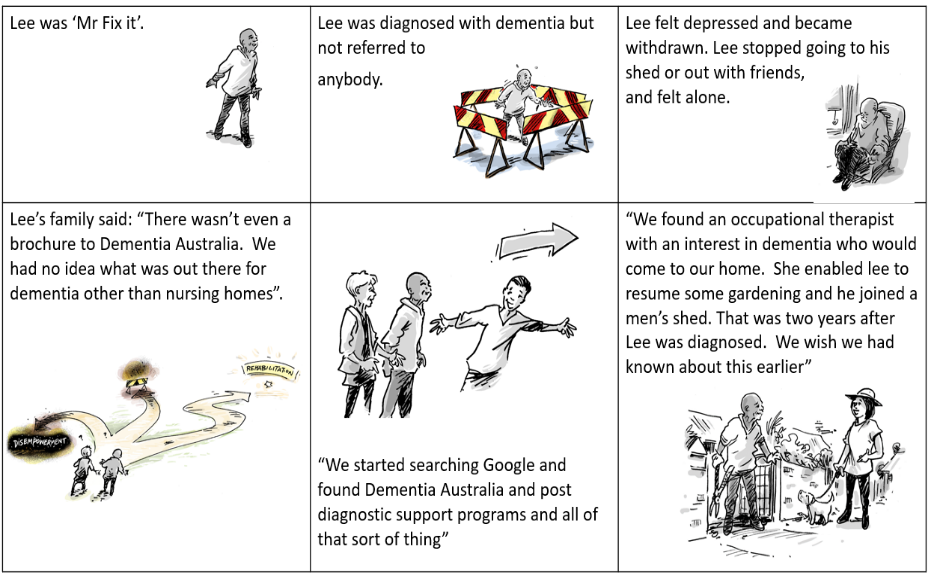 |
| **Briefing Note Workshop 3** | Briefing Note   - What we know from Workshops 1 and 2 - Suggested solutions - Preparation for Workshop 3 – refining the solutions - After Workshop 3 – what’s next?   During Workshop 3, the project team shared the proposed INCLUDE solutions for feedback. |
| **Summary of workshop 3** | Workshop 3 summary was sent after workshop 3 |
